# Properties of Coatings Based on Calcium Phosphate and Their Effect on Cytocompatibility and Bioactivity of Titanium Nickelide

**DOI:** 10.3390/ma16072581

**Published:** 2023-03-24

**Authors:** Ekaterina S. Marchenko, Gulsharat A. Baigonakova, Kirill M. Dubovikov, Oleg V. Kokorev, Ivan I. Gordienko, Ekaterina A. Chudinova

**Affiliations:** 1 Laboratory of Superelastic Biointerfaces, National Research Tomsk State University, 36 Lenin Ave., 634045 Tomsk, Russia; 2 Department of Pediatric Surgery, Ural State Medical University, 620014 Yekaterinburg, Russia

**Keywords:** titanium nickelide, coating, surface, calcium phosphate, bioactivity, cytocompatibility, structure

## Abstract

Coatings based on calcium phosphate with thicknesses of 0.5 and 2 μm were obtained by high-frequency magnetron sputtering on NiTi substrates in an argon atmosphere. The coating was characterized using X-ray diffraction, scanning electron microscopy, atomic force microscopy, and in vitro cytocompatibility and bioactivity studies. A biphasic coating of tricalcium phosphate (Ca_3_(PO_4_)_2_) and hydroxyapatite (Ca_10_(PO_4_)_6_(OH)_2_) with a 100% degree of crystallinity was formed on the surface. The layer enriched in calcium, phosphorus, and oxygen was observed using scanning electron microscopy and energy-dispersive X-ray spectroscopy. Scanning electron microscopy showed that the surface structure is homogeneous without visible defects. The 2 µm thick coating obtained by sputtering with a deposition time of 4 h and a deposition rate of 0.43 µm/h is uniform, contains the highest amount of the calcium phosphate phase, and is most suitable for the faster growth of cells and accelerated formation of apatite layers. Samples with calcium phosphate coatings do not cause hemolysis and have a low cytotoxicity index. The results of immersion in a solution simulating body fluid show that NiTi with the biphasic coating promotes apatite growth, which is beneficial for biological activity.

## 1. Introduction

At present, the development and research of bioactive calcium phosphate coatings on metal medical alloys is of high practical importance [1,2,3]. Uneven tissue growth and poor osteoconductivity characteristics on the metal surfaces of implants due to changes in surface energy are the main factors contributing to the failure of most metal implants. Calcium phosphate coatings enable the accelerated integration of implants into the body due to rapid deposition and the growth and strong adhesion of cells to surrounding tissues compared to bioinert metal surfaces of implants [4,5,6]. It is known that the release of calcium and phosphate ions contributes to the deposition of carbonate-apatite on the surface of the implant, which is a matrix for the adhesion and reproduction of cells that form bone tissue [4,5,6,7,8,9]. The modified surface rapidly absorbs more protein and promotes cell adhesion, especially osteoblasts, which accelerates the formation of chemical bonds between the bone and the implant [10].

Material for the replacement of bone defects must meet certain criteria, such as biocompatibility, osteoconductivity, osteoinductivity, porosity, mechanical stability, safety, and economy [11]. The properties of coatings depend not only on the choice of materials but also on the method and conditions of their deposition. Basic characteristics of coatings such as crystallinity, phase composition, morphology, and structure should be taken into account. The phase composition and crystal structure on the surface of the coating determine the rate and kinematics of the formation of natural apatite upon contact with body fluids [12]. Coatings that are a mixture of two different compositions and/or phase calcium phosphates have an advantage [13]. The most common combinations of calcium phosphate materials are HAP+β-TCP [13,14,15,16], HAP+α-TCP [17,18,19], and α-TCP+β-TCP [20,21,22,23].

Studies on the use of calcium phosphate coatings on nickel–titanium and titanium alloy surfaces for biomedical applications show that they have a positive effect on the biological activity of implants in terms of both their physical properties and their ability to integrate with the surrounding bone tissue [2,24,25]. In [26], the function of a CaP coating on a titanium substrate was to prevent fibrous tissue encapsulation at the titanium–bone interface and to increase the osteoconductivity of titanium. In [27], a calcium phosphate coating on nickelide titanium alloy improved its biocompatibility and mechanical properties, making it a promising candidate for biomedical applications. Calcium-phosphate-coated NiTi has improved in vitro bioactivity and electrochemical behavior and is more effective in suppressing nickel ion leaching than uncoated and thermally oxidized NiTi [28,29]. Hydroxyapatite crystals formed on the coated NiTi surface after immersion in artificial body fluid for 7 days, confirming its surface bioactivity [30]. In [31], the authors showed that a hydroxyapatite coating on porous NiTi alloys has excellent stability and an increased rate of apatite deposition, and the rate of Ni ion release through coatings on alloys with different porosity ratios in the simulated body fluid is markedly reduced compared to uncoated alloys. Additionally, the inner surface of the porous nickel–titanium alloy has an increased rate of apatite deposition due to the rough surface. A biocompatibility study of CaP coatings deposited by pulsed electrodeposition on a superelastic NiTi alloy demonstrated a high cell density and high proliferation after 5 days and a hydrophilic surface [32]. The crystallization rate of calcium phosphate compounds and the stability of coatings in biological environments depend on the morphology and structure of the coatings.

This study is aimed at studying the structure and phase composition of coatings based on calcium phosphate for the cytocompatibility and bioactivity of titanium nickelide materials. NiTi alloys have shape memory and superelasticity effects and can be reversibly deformed up to 8% [33,34]. The protective oxide film spontaneously formed on the surface of NiTi increases its biological inertness and corrosion resistance [35,36]. However, monolithic NiTi alloys exhibit insufficient bioactivity and an inadequate friction coefficient, which indicates the need for additional modification of their surface. Therefore, increasing the biointegration capacity and biological activity of metal implants is an important area of research. The method of high-frequency magnetron sputtering used in this work makes it possible to deposit uniform coatings with high adhesion and uniform thickness and composition.

## 2. Materials and Methods

### 2.1. Deposition of Coating

Titanium nickelide substrates were fabricated in an induction furnace ISV-0.004 PI M1 (Tula, RF) by remelting spongy titanium and nickel grade H1 with the addition of a molybdenum dopant according to the scheme Ti_50_Ni_49.7_Mo_0.3_. Molybdenum was added for manufacturability and machinability.

The coatings were deposited using the COMPLEX universal installation developed at the Institute of High Current Electronics of the Siberian Branch of the Russian Academy of Sciences (HCEI SB RAS), which is included in the list of unique scientific installations of the Russian Federation as part of the UNIQUUM complex. Coatings were deposited using the method of plasma-assisted high-frequency (HF) sputtering of powder targets developed at the Institute of Laser and Energy Emissions of the HCEI SB RAS. A target with a diameter of 200 mm without a magnetic system was used. Compacted powders of calcium phosphates were used as targets, the properties of which are given in Table 1.

**Figure 1:**
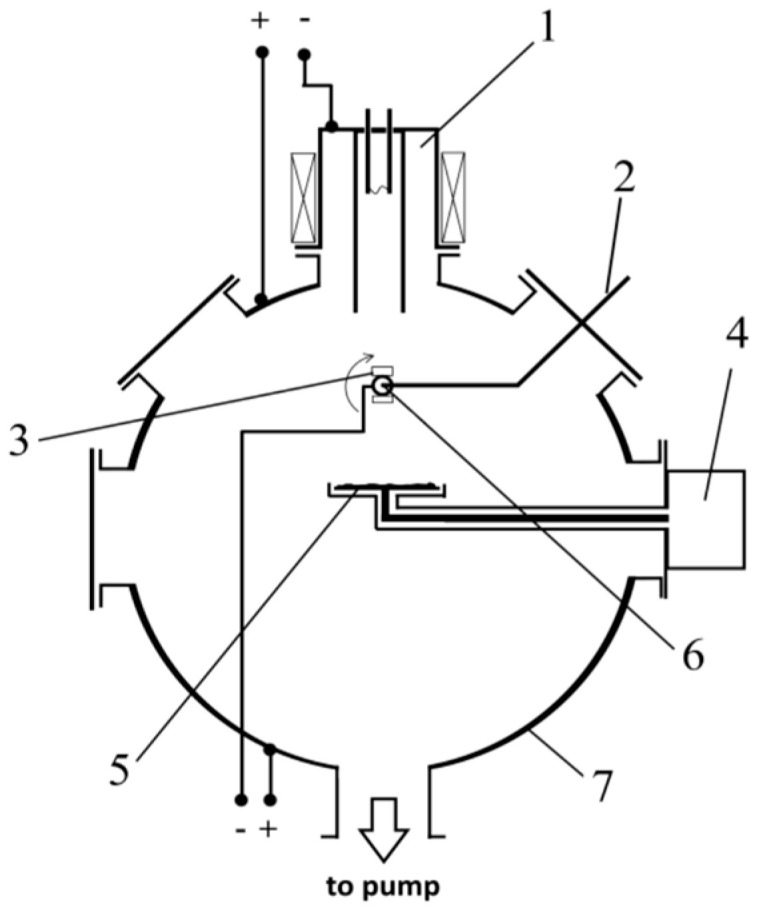
Scheme of the discharge system for ion-plasma HR deposition of coatings. 1—Plasma generator; 2—thermocouple; 3—substrate for deposition; 4—HR generator; 5—target; 6—holder for the substrate; 7—vacuum chamber.

**Figure 2:**
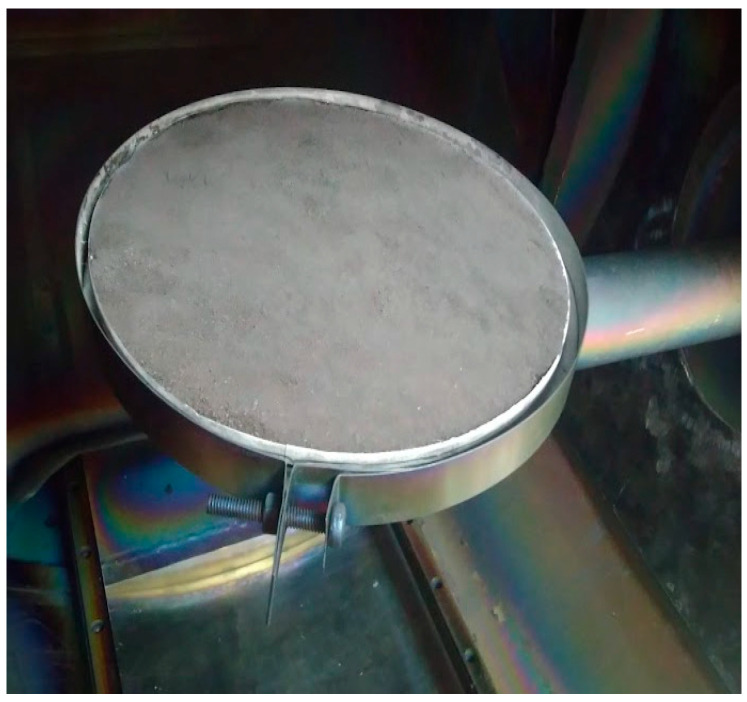
The appearance of the discharge system during the deposition of a film based on calcium phosphates.

To intensify the process of spraying the powder of calcium phosphates, the gas plasma generator “PINK” was used. It contributed to the creation of volumetric argon plasma in a vacuum chamber. When an HR potential was applied to the target, argon ions were extracted from the plasma and bombarded the target, resulting in the intense sputtering of calcium phosphate powder. The scheme of the discharge system for the deposition of coatings based on calcium phosphate powder is shown in Figure 1, and the appearance is shown in Figure 2.

The technological process of coating deposition consists of the following stages. The sample and the target for sputtering are placed in a vacuum chamber, and the pressure is reduced to 5 × 10^–3^ Pa. Argon is supplied to the chamber up to a pressure of 0.1–0.4 Pa, the plasma generator is turned on, and a negative bias voltage is applied to the substrate to clean and activate the surface with argon plasma. After surface treatment, the RF generator connected to the target is turned on to initiate the material sputtering process. The formation of a coating on the surface of the substrate occurs as a result of applying a bias voltage to it.

The current–voltage characteristics of a non-self-sustained HR discharge were studied with variations in the pressure of the working argon gas in the range from 0.1 to 0.5 Pa, the discharge current of the PINK plasma generator from 5 to 70 A, and the HR power supplied to the target from 300 to 1000 W. According to the set of parameters, an argon pressure of 0.3 Pa, a PINK plasma generator discharge current of 30 A, and an HR power of 600 W on the target were chosen as the optimal parameters for the process of depositing calcium phosphates.

In this mode, the rate of deposition of a film based on calcium phosphates is 0.5 µm/h, which ensures the stable operation of the PINK plasma generator, no overheating of the target, and a negative auto-bias on the target of 800–1100 V, which is optimal for sputtering the material and does not damage the HR-input insulators.

With an increase in the deposition time from more than 4 h up to 6 h, no increase in the coating thickness was observed on the samples. The maximum obtained coating thickness was 2 μm. It was not possible to increase the thickness of the deposited coatings, most likely due to the spraying of the coating with the gas plasma.

To obtain the optimal coating thickness of 2 µm, sputtering processes were carried out with a deposition time of 4 h and a deposition rate of 0.43 µm/h.

Table 2 shows the coating thickness depending on the duration of the process and the deposition rate.

For this study, NiTi samples with 0.5 and 2.0 µm thick calcium phosphate coatings were obtained.

### 2.2. Surface Characterization Methods

The surface morphology of the coatings was characterized using a Axia ChemiSEM Scanning Electron Microscope (Thermo Fisher Scientific, Waltham, MA, USA) with an elemental composition microanalyzer.

The phase composition and crystallinity of the coated samples were studied using X-ray diffraction on the XRD-6000 diffractometer with Cu Kα radiation (Shimadzu, Japan) in the conventional Bragg–Brentano geometry. The amounts of phases present in the coating were estimated by full-profile Rietveld analysis using the POWDER CELL 2.4 software and the PDF4+ crystal structure database.

The surface topography of the synthesized coatings was studied by atomic force microscopy (AFM) using an NT-MDT scanning probe microscope (NT-MDT, Moscow, RF) with a SOLVER HV vacuum chamber in semi-contact mode. Data processing was carried out using the Gwyddion program.

### 2.3. In Vitro Testing

The cytocompatibility of the coatings was evaluated in vitro using a culture viability test of rat bone marrow mesenchymal stem cells after 72 h of cultivation. For this study, 3 samples were used. The size of samples for the test was 10 mm × 10 mm × 10 mm. Before applying the cell suspension, the samples were degreased with 70% ethanol, washed in an ultrasonic bath, and autoclaved at 180 °C for one hour. Rat bone marrow mesenchymal stem cells were cultured with samples in a CO_2_ incubator for 72 h under standard conditions at 37 °C in 5% CO_2_ and a humidified atmosphere. The culture medium consisted of DMEM/F12 (Paneco, RF) supplemented with 10% fetal bovine serum, 40 μg/mL gentamicin, and 250 mg/L glutamine. The matrices were studied during static cultivation in a standard 12-well culture dish.

The percentage of cytotoxicity is the average of triplicates and is calculated as:

$$\mathrm{Cytotoxicity}\;\mathrm{Index},\;(\%)=\frac{O-K}{O}\;\times \;100\%,$$

where O is the extinction index of the experimental sample, and K is the index of extinction of the control sample.

To determine the hemolysis index, blood from a healthy volunteer donor was mixed with a solution containing sodium citrate (3.8 wt.%). It was then diluted 9:1 with saline (4:5 ratio by volume).

The samples were immersed in a standard tube containing 10 mL of physiological solution, which was preincubated at 37 °C for 30 min. Then, 0.2 mL of diluted blood was added to this standard tube, and the resulting mixture was incubated for 60 min at 37 °C. Similarly, 0.85% NaCl physiological saline was used as a negative control, and deionized water was used as a positive control. After that, all tubes were centrifuged for 5 min at 3000 rpm, and then the supernatant was transferred to a cuvette for spectroscopic analysis at a wavelength of −545 nm. Hemolysis was calculated using an ultraviolet spectrophotometer at 545 nm (Picon, Uniplan, Russia). To evaluate the biological activity of calcium phosphate coatings on TiNi substrates, samples were immersed in a solution that imitated the chemical composition of fluids in the body with a pH close to that of human blood plasma. After the solution was prepared, it was immediately heated to 37.5 °C, and carbogen gas was added to adjust the pH to 7.35, thus preventing sediment formation. The composition of the liquid was constantly adjusted by the automatic addition of distilled water. On day 30, the samples were removed and placed in an ultrasonic bath to remove any debris and dried at 180 °C for 2 h. The bioactivity of the coating was assessed by the formation of apatite layers during its immersion in the solution. The change in the volume fraction was determined from X-ray diffraction patterns.

## 3. Results and Discussion

### 3.1. Structure and Phase Composition of Coatings

**Figure 3:**
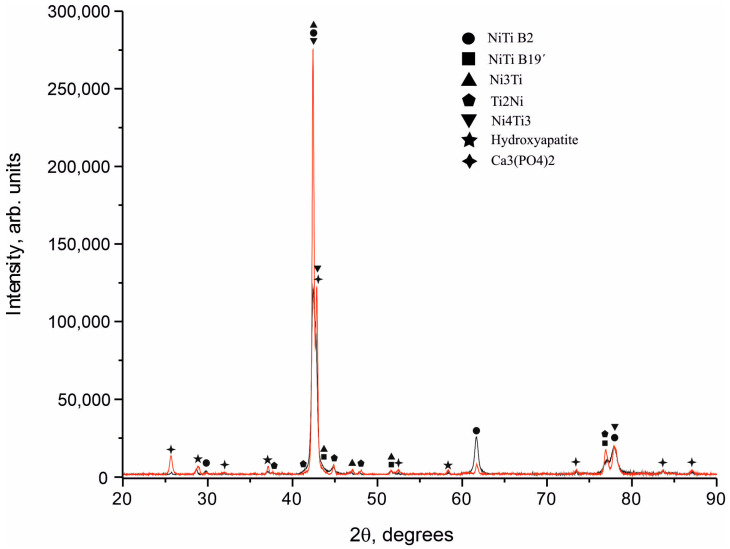
X-ray diffraction patterns of NiTi samples coated with hydroxyapatite targets, where the black line is sample 1, and the red line is sample 2.

Diffraction spectra were obtained from NiTi samples coated with calcium phosphate targets (Figure 3). All samples were in a mixed structural state and consisted mainly of the TiNi phase in two crystallographic modifications of B2 austenite and B19’ martensite. In addition to the main structural lines, reflections from intermetallic phases of Ti_2_Ni and Ni_3_Ti were found in the spectrum. Coatings were formed as a mixture of two phases, Ca_3_(PO_4_)_2_ and Ca_10_(PO_4_)_6_(OH)_2_. The phases of tricalcium phosphate (Ca_3_(PO_4_)_2_) and hydroxyapatite (Ca_10_(PO_4_)_6_(OH)_2_) were found on the surfaces of all samples with a 100% degree of crystallinity. Amorphous X-ray scattering was not found in the X-ray diffraction pattern in the region of structural reflections. The high intensity of the reflection from the phase of tricalcium phosphate (Ca_3_(PO_4_)_2_) indicates its predominant content in the coating. Thus, Ca_3_(PO_4_)_2_ is the main structural component, and HA is a minor phase in the coating.

It is known that hydroxyapatite (Ca_10_(PO_4_)_6_(OH)_2_) and β-tricalcium phosphate (Ca_3_(PO_4_)_2_) are the basis of the mineral part of human bone and are characterized by high biological activity [37,38]. β-Tricalcium phosphate promotes the proliferation of osteogenic progenitor cells due to the nanoporous structure [39,40]. Hydroxyapatite is more stable compared to β-tricalcium phosphate but decomposes more slowly [10]. The advantages of a two-phase coating of hydroxyapatite and β-tricalcium phosphate include better biocompatibility with the surrounding bone tissue, improved osseointegration, and improved mechanical stability of implantable devices [41,42]. The better solubility of β-tricalcium phosphate in physiological environments and the slower degradation rate of hydroxyapatite make the combination of the two ideal for biomedical applications. The two-phase coating has also been shown to enhance bone cell attachment and proliferation. Additionally, the combination of the two phases can provide a more gradual gradient between the implant surface and the surrounding tissue, which can potentially reduce the risk of inflammation and implant failure. Experimental results have shown that such biphasic coatings have a higher ability to adsorb fibrinogen, insulin, or collagen than pure hydroxyapatite [43]. Biphasic calcium phosphates were evaluated mainly in terms of biological activity, bioresorbability, and osteoinductivity [44]. These coatings have been used and studied as bone grafts, bone substitutes, and dental materials [13,45]. A mixture of hydroxyapatite and β-tricalcium phosphate is actively used to stimulate the osteogenic differentiation of mesenchymal stem cells, increase cell adhesion, attach growth factors, and improve mechanical properties [46,47]. In [48], the percentage of hydroxyapatite and tricalcium phosphate in the coating was varied to determine the best biocompatibility and antibacterial properties. It was shown that the sample consisting of hydroxyapatite and β- tricalcium phosphate at a ratio of 1:1 showed a significant increase in bioactivity and antibacterial properties compared to the uncoated titanium control sample. It should be noted that the high crystallinity of the calcium phosphate coating with a nano-rough surface has a positive effect on cell adhesion, in contrast to amorphous ones [38].

**Figure 4:**
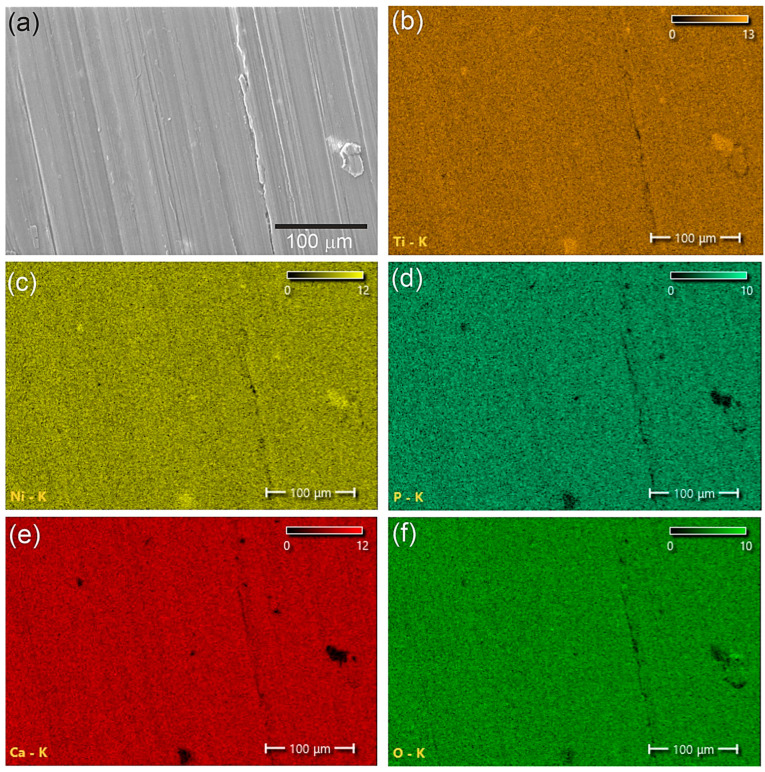
SEM image (**a**) and EDS mapping of the element composition (**b**–**f**) of a coating of sample № 1.

**Figure 5:**
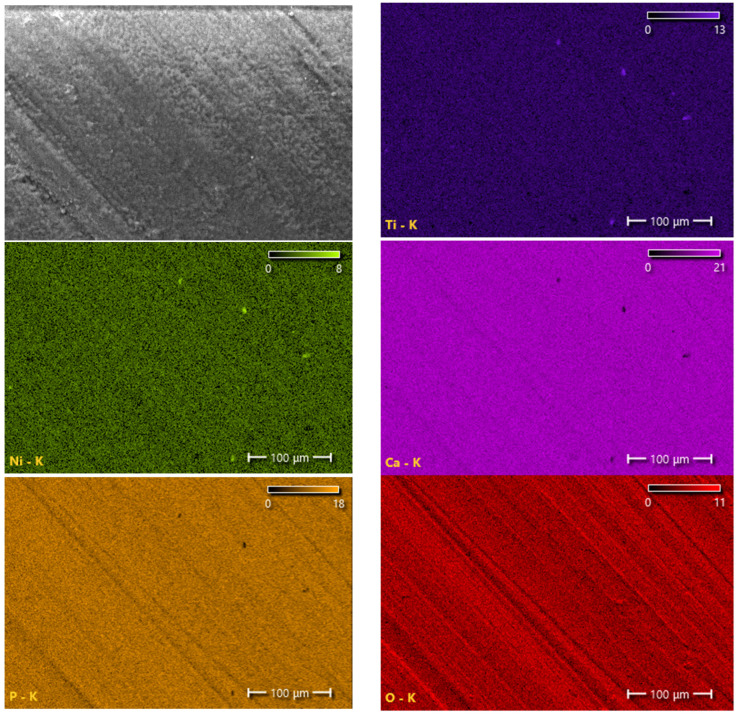
SEM image and EDS mapping of the element composition of a coating of sample № 2.

Scanning electron microscopy and element distribution maps showed that the two-phase coatings of calcium phosphate and hydroxyapatite are fairly uniform and dense and have practically no pores (Figure 4 and Figure 5). The distribution of elements in the coating was determined using energy-dispersive X-ray spectroscopy (EDS). The average contents of elements in a coating with a thickness of 0.5 μm were 57 at.% oxygen, 5 at.% phosphorus, 9 at.% calcium, 16 at.% titanium, and 13 at.% nickel. The average contents of elements in a 2 μm thick coating were 57 at.% oxygen, 12 at.% phosphorus, 24 at.% calcium, 4 at.% titanium, and 3 at.% nickel.

**Figure 6:**
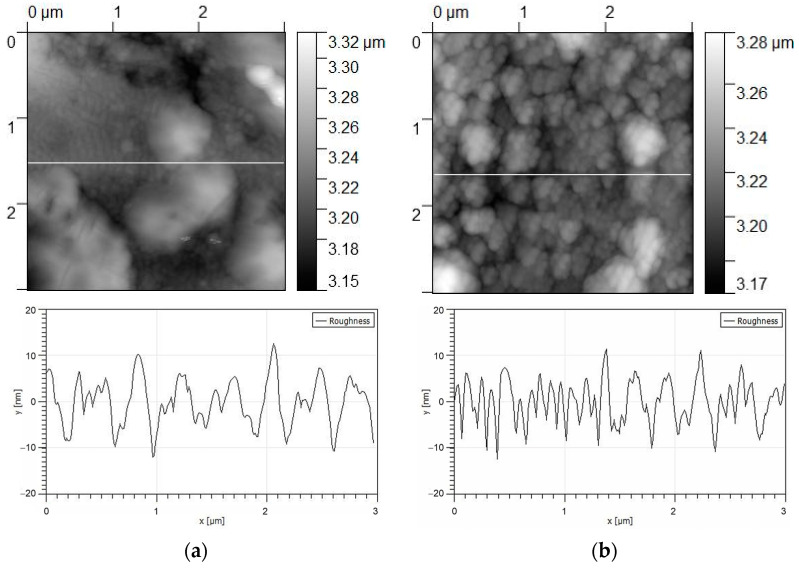
Surface topography of coated samples № 1 (**a**) and № 2 (**b**).

The surface topography of the coatings was studied by atomic force microscopy (Figure 6). The coating surface with a thickness of 0.5 µm is characterized by a grainy rough morphology with an average roughness parameter of 4.02 ± 2.3 nm and a grain size of 0.1 to 0.5 µm. The coating surface with a thickness of 2 μm has an average roughness parameter of 1.85 ± 1.6 nm and acquires an island topography with a size of 0.5 to 1 μm formed by coagulated grains.

### 3.2. Cytocompatibility and Bioactivity of Coatings

In vitro studies were carried out on the hemolysis of erythrocytes on nickel–titanium samples with calcium phosphate coatings with various thicknesses. The percentage of hemolysis was calculated by the formula:

$$\mathrm{Hemolysis},\;(\%)=\frac{\left(\mathrm{OD}\;\left(\mathrm{test}\right)-\;\mathrm{OD}\;\left(\mathrm{negative}\;\mathrm{control}\right)\right)\;}{(\left(\mathrm{OD}\;\left(\mathrm{positive}\;\mathrm{control}\right)-\;\mathrm{OD}\;\left(\mathrm{positive}\;\mathrm{control}\right)\right)}\times 100\%,$$

where OD (test) is the extinction index of the experimental sample, OD (negative control) is the extinction index of the negative control, and OD (positive control) is the extinction index of the positive control.

**Figure 7:**
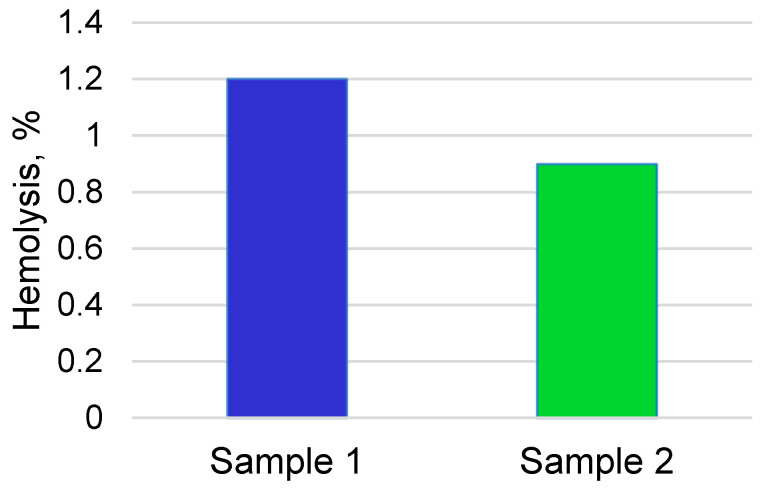
The percentage of hemolysis of samples with coatings with different thicknesses.

An assessment of the hemolytic effect of alloy samples with coatings of different thicknesses is shown in Figure 7.

In these studies, it was noted that samples with calcium phosphate coatings with thicknesses of 0.5 and 2 μm showed erythrocyte hemolysis amounts of 1.2 ± 0.2% and 0.9 ± 0.15%, respectively, which are not high degrees of hemolysis and are consistent with the standards developed in the National State Standard and ISO standards for biocompatible materials in contact with the circulatory system (Figure 7).

**Figure 8:**
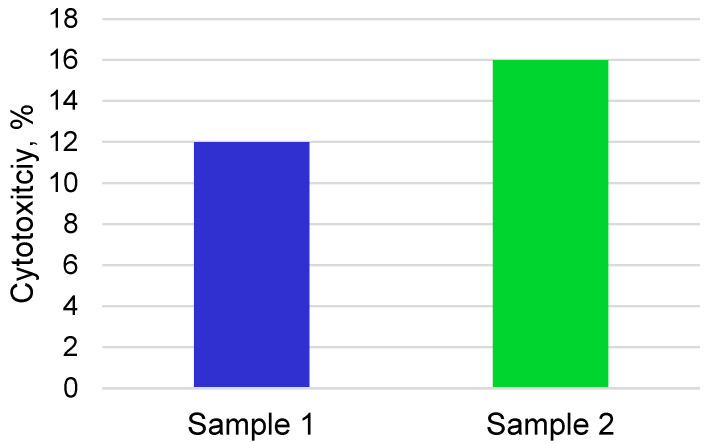
Cytotoxicity indices of samples of porous titanium nickelide alloys obtained in closed and open flow reactors, tested in mesenchymal stem cells after 24 h.

The study of the cytotoxicity of samples in mesenchymal stem cells using the MTT test after 24 h showed that the two series of samples have low cytotoxicity indices of 12 ± 2.3% and 16 ± 3.1%, respectively (Figure 8).

**Figure 9:**
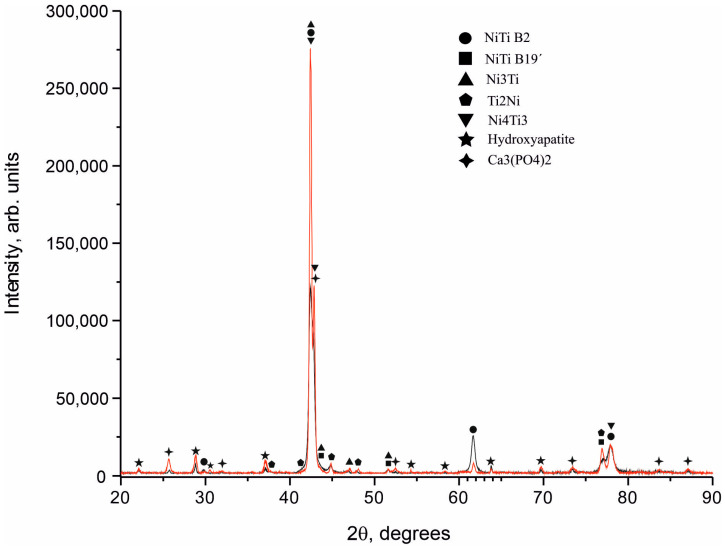
XRD pattern after immersing samples in Ringer’s solution, where the black line is sample 1, and the red line is sample 2.

After immersing samples of coatings based on calcium phosphate in Ringer’s solution, an increase in the height of the structural lines of the phases of hydroxyapatite (Ca_10_(PO_4_)_6_(OH)_2_) was found in X-ray diffraction patterns (Figure 9).

**Figure 10:**
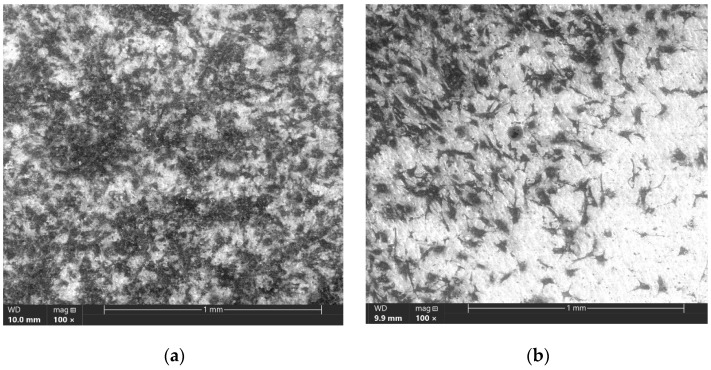
SEM images of coated samples № 1 (**a**) and № 2 (**b**) after 30 days of immersion in a solution simulating body fluid.

The number of viable, adherent cells on the surface of the 0.5 µm coating is much greater and covers more than 70% of the surface compared to the sample with a coating thickness of 2 µm. However, the 2 μm thick coating showed the accelerated formation of apatite layers, which is evidenced by a large proportion of hydroxyapatite areas (Figure 10).

## 4. Conclusions

As a result of this research, the optimal modes for applying coatings based on calcium phosphates were obtained. For the study, NiTi samples with 0.5 and 2.0 µm thick calcium phosphate coatings were obtained. The qualitative phase composition and the corresponding crystallinity of the coating do not change depending on the deposition regime. Coatings are formed as a mixture of two phases, Ca_3_(PO_4_)_2_ and Ca_10_(PO_4_)_6_(OH)_2_, where tricalcium phosphate is the main structural component, and HA is a minor phase in the coating. A 2.0 µm thick coating obtained by sputtering with a deposition time of 4 h and a deposition rate of 0.43 µm/h contains the largest amount of the calcium phosphate phase and is more suitable for the faster growth and strong attachment of cells. In vitro studies show that both coatings have a low cytotoxicity index, do not cause a high degree of hemolysis, and are preferred for cell growth. The bioactivity study shows that when immersed in a simulated body fluid, the coating obtained at a deposition rate of 0.43 µm/h and a deposition time of 4 h has a more accelerated tendency to build up hydroxyapatite on its surface.

## Figures and Tables

**Table 1:** Quality indicators of calcium phosphate powder.

№	Name of Indicator	Specification Requirements in GB 1889–2004/FCC	Analysis Result
1.	Appearance	Homogeneous, white crystalline powder, tasteless and odorless	Conforms
2.	Mass fraction of 2-water disubstituted potassium phosphate (CaHPO_4.2_H_2_O), %, not less than	98.5	99.58
3.	Content CaO, %	31.4–32.9	32.4
4.	Degree of whiteness (WG), %, not less than	95	96.12
5.	Dispersion		
	(Sieve 325 mix), %, not less	99.0	99.6
	(Sieve 200 mix), %, not less	99.95	99.96
6.	Mass fraction of fluorides, mg/kg, no more than	50	9
7.	Mass fraction of chlorides, mg/kg, no more than	300	<100
8.	Mass fraction of losses during annealing, %, within	24.5–26.5	26.1

**Table 2:** The deposition regime of calcium phosphate coating.

Sample Number	Deposition Time, h	Thickness, µm	Deposition Rate, µm/h
1	1	0.5	0.5
2	4	2	0.43

## Data Availability

Not applicable.
